# Regulation of Th1 T Cell Differentiation by Iron *via* Upregulation of T Cell Immunoglobulin and Mucin Containing Protein-3 (TIM-3)

**DOI:** 10.3389/fimmu.2021.637809

**Published:** 2021-05-24

**Authors:** Christa Pfeifhofer-Obermair, Piotr Tymoszuk, Manfred Nairz, Andrea Schroll, Gloria Klais, Egon Demetz, Sabine Engl, Natascha Brigo, Günter Weiss

**Affiliations:** ^1^ Department of Internal Medicine II, Medical University of Innsbruck, Innsbruck, Austria; ^2^ Department of Biotechnology & Food Engineering, MCI-The Entrepreneurial School, Innsbruck, Austria; ^3^ Christian Doppler Laboratory for Iron Metabolism and Anemia Research, Medical University of Innsbruck, Innsbruck, Austria

**Keywords:** iron, T helper cell, TIM-3, immune checkpoint inhibitors, interferon-gamma, intracellular bacteria, infection control

## Abstract

Iron plays an important role in host–pathogen interactions, in being an essential element for both pathogen and host metabolism, but also by impacting immune cell differentiation and anti-microbial effector pathways. Iron has been implicated to affect the differentiation of T lymphocytes during inflammation, however, so far the underlying mechanism remained elusive. In order to study the role of iron in T cell differentiation we here investigated how dietary iron supplementation affects T cell function and outcome in a model of chronic infection with the intracellular bacterium *Salmonella enterica serovar typhimurium* (*S. Typhimurium*). Iron loading prior to infection fostered bacterial burden and, unexpectedly, reduced differentiation of CD4^+^ T helper cells type 1 (Th1) and expression of interferon-gamma (IFNγ), a key cytokine to control infections with intracellular pathogens. This effect could be traced back to iron-mediated induction of the negative immune checkpoint regulator T cell immunoglobulin and mucin domain-containing protein 3 (TIM-3), expressed on the surface of this T cell subset. *In vitro* experiments demonstrated that iron supplementation specifically upregulated mRNA and protein expression of TIM-3 in naïve Th cells in a dose-depdendent manner and hindered priming of those T cells towards Th1 differentiation. Importantly, administration of TIM-3 blocking antibodies to iron-loaded mice infected with *S. Typhimurium* virtually restored Th1 cell differentiation and significantly improved bacterial control. Our data uncover a novel mechanism by which iron modulates CD4^+^ cell differentiation and functionality and hence impacts infection control with intracellular pathogens. Specifically, iron inhibits the differentiation of naive CD4^+^ T cells to protective IFNγ producing Th1 lymphocytes *via* stimulation of TIM-3 expression. Finally, TIM-3 may serve as a novel drug target for the treatment of chronic infections with intracellular pathogens, specifically in iron loading diseases.

## Introduction

Because of its high redox activity, iron is a key component of several enzymatic processes. Virtually every cell of the body requires iron for metabolism and proliferation. In mammals most iron for the daily needs is deliverd by macrophages which ingest aged or damaged red blood cells (1). After phagocytosis iron is extracted from erythrocyte heme and exported from the macrophage *via* the iron-exporter ferroportin-1 to the circulation, where it is bound to transferrin, and taken up by cells *via* transferrin receptor-1 (TfR-1) (2). This uptake is thus of high relevance for the differentiation of most cells including lymphocytes (3, 4). Since iron is crucial for both microbes and mammalian cells, iron homeostasis undergoes subtle changes during infection and inflammatory processes which is mediated by various mechanisms (5). Moreover, iron availability affects lymphocyte proliferation and function (6–8). In human T lymphocytes, iron induces refractoriness to IFNγ/STAT1 (Signal transducer and activator of transcription 1) signaling *via* involvement of the TfR-1 (9). The TfR-1 regulates IFNγ signaling in activated T cells by interacting with the T cell receptor (TCR) after being recruited to the immunological synapse in response to TCR activation (10). Activation of the TCR causes naïve CD4^+^ T cells to proliferate and differentiate into different subsets [Th type 1 (Th1) cells, Th2, Th17, Th9, Th22, and regulatory T cells (Treg)] (11), depending on the specific cytokine milieu.

As a consequence of persisting antigenic stimulation and inflammation, like in chronic infections and cancer, T cell exhaustion develops as a state of cellular and immunological dysfunction (12). Exhausted T cells are characterized by reduced cytokine production and over-expression of a distinct set of inhibitory receptors like programmed cell death protein 1 (PD-1), cytotoxic T-lymphocyte-associated Protein 4 (CTLA-4), Lymphocyte-activation gene 3 (Lag-3), and T cell immunoglobulin and mucin containing protein-3 (TIM-3) (12). Several studies demonstrated a key role of TIM-3 in T cell dysfunction and exhaustion (13–15) and in activating signaling cascades leading to the regulation of immune cell functions (16–20).

Whereas inhibitory receptors on lymphocytes such as PD-1, CTLA-4, Lag-3, T cell immunoreceptor with Ig and ITIM domains (TIGIT-1), and TIM-3 have been identified as crucial components mediating T cell exhaustion in cancer and viral infections (21), the role in chronic bacterial infections is largely elusive. However, upon infection with *Mycobacterium tuberculosis Tim*-3 knockout mice show an improved survival (22), pointing to the importance of TIM-3 in bacterial infections.

*Salmonella enterica serovar* T*yphimurium* (*S. Typhimurium*) is a Gram negative, facultative intracellular bacterium which causes typhoid fever like infections in mice. Many studies with attenuated bacterial strains have emphasized an essential role for *Salmonella*-specific CD4^+^ Th1 cells as important host response mechanisms to intracellular pathogens (23, 24). Of note, *Salmonella* are siderophilic bacteria and iron loading is associated with increased bacterial multiplication and impaired immune control of infection (25).

Here, we provide evidence that detrimental effects of high systemic iron on the integrity of bacterial immune defense in a model of chronic *S. Typhimurium* infection can be traced back to a strong iron- mediated upregulation of TIM-3 expression in helper T cells and an impaired differentiation into protective IFNγ producing Th1 lymphocytes. Administration of TIM-3 blocking antibodies to iron-loaded animals restored the Th1 cell expansion and greatly reduced bacterial burden, indicating that the negative checkpoint modulator acts as a crucial, iron-dependent regulator of T cell immune response to bacterial pathogens.

## Materials and Methods

### Mice

C57BL/6 mice had free access to food and water and were housed according to institutional and governmental guidelines in the animal facility of the Medical University of Innsbruck with a 12-hour light-dark cycle and an average temperature of 20°C ± 1°C. Animal experiments were approved by the Austrian Federal Ministry of Science and Research (licence number BMWF-66.011/0113-WF/V/3b/2016) according to the directive 2010/63/EU. Nramp^G169^ C57BL/6 mice are a kind gift from Ferric C. Fang (University of Washington, Seattle). All experiments were performed with male mice.

### Infection of Mice

Wildype *Salmonella enterica* serovar *typhimurium* (*S. Typhimurium*) strain ATCC14028 was used for experiments and grown under sterile conditions in LB broth (Sigma-Aldrich) to late-logarithmic phase. For the infection model male mice were fed with different iron diets for two weeks and during the course of the infection. Low iron diet had an iron content of ≤9 mg iron/kg diet (26), high ion diet contained 5 g iron/kg diet (both diets from Altromin). Male mice were used at 8–12 weeks of age and infected intraperitoneally with 500 CFU of S*. Typhimurium* in 200 μl PBS (27–29). After 14 days of infection mice were euthanized by cervical dislocation, spleens and livers were isolated, erythrocytes lysed, and flow cytometry was performed. The bacterial load of organs was determined by plating serial dilutions of organ homogenates on LB agar (Sigma-Aldrich) under sterile conditions and the number of bacteria calculated per gram of tissue.

### Blocking Antibody Experiments

Mice were fed and infected as described. Additionally from the day of infection on, mice were intraperitoneally injected with 100 µg inVivo MAb anti-mouse TIM-3 antibody (BioXCell; BE0115) or InVivoMAb rat IgG2a isotype control (BioXCell; BE0089) (30–34) in 200 µl PBS every second day till day 14 post infection.

### Flow Cytometry Analysis

Spleens were homogenized through 100 µm nylon cell strainer (Falcon) and red blood cells were lysed by incubation in ACK buffer (150 mM NH_4_Cl, 10 mM KHCO_3_, 0.1 mM Na_2_EDTA) for 2 min at room temperature.

Flow cytometry staining was performed with panels of antibodies specific for naïve, activated/memory and exhausted T cells (anti-CD45-FITC, anti-CD3-Biotin + Streptavidin-PeCy7, anti-CD4-FITC, anti-CD62L-PeCy7, anti-CD44-APC, anti-TIM-3-APC, anti-PD1-PE) and neutrophils, macrophages and monocytes (anti-CD45-FITC, anti-F4/80-BV421, anti-CD11b-APC, anti-Ly6G-PerCPeF710, anti-MerTK-PECy7, anti-Ly6C-BV510), in PBS with 0.5% FCS 2mM EDTA for 15 min. For the staining of iron recptors anti-TfR-1/CD71-PE, anti-ZIP14 + donkey anti-rabbit DyLight488, and anti-DMT1 + donkey anti-rabbit DyLight488 were used. For intracellular staining cells will be stimulated with a mix containing 10 µg/ml Brefeldin A (Sigma), 50 ng/ml PDBu (Sigma) and 500 ng/ml ionomycin (Sigma) in RPMI-1640 (PAN Biotech) plus 10% FCS (Biochrom) plus 1% penicillin/streptomycin (Lonza) plus 2 mM L-glutamine (Lonza) for 4 h. Brefeldin A leads to blockade of protein transport to the Golgi complex and therefore the accumulation of proteins in the endoplasmic reticulum. Following, cytokines are trapped inside the cells and can be detected by intracellular staining as described. The cells were then formalin-fixed, permeabilized (0.05% Triton X-100 in PBS) and stained for cytokines (anti-IFNγ-PE, anti-IL17-FITC, anti-IL4-PE), and transcription factors (anti-FOXP3-FITC) for 1 h. All antibodies were from Biolegend. Cells were analyzed with Gallios and Cytoflex S flow cytometers (Beckman Coulter) and FlowJo Software (Beckton Dickinson).

### Iron Measurement

Serum iron concentrations are measured with a colorimetric iron quantification kit (QuantiChrom Iron Assay Kit, BioAssay Systems) following the manufacturer’s instructions. Tissue iron was quantified using a colorimetric method with bathophenanthroline disulfonic acid (35). In brief, organ lysates were hydrolyzed with acid for 24 h at 65°C, mixed with a colorimetric solution containing sodium acetate, bathophenanthroline disulfonic acid and l-ascorbic acid and absorbance at 539 nm was measured. The iron content of the organ was calculated from a standard curve and normalized to the protein content of the lysate determined by the Bradford method.

### Splenocyte Cell Culture

Spleens were isolated and after lysis of erythrocytes using the Mouse Erythrocyte Lysing Kit (R&D Systems) 2.5 x 10^5^ splenocytes per well were then seeded in a 96-well round bottom plate and stimulated with 4 µg/ml plate-bound or 1 µg/ml soluble rat anti-mouse CD3 (clone 17A2; BD Pharmingen). Ferric chloride FeCl_3_ (Sigma Aldrich), ferric sulfate Fe_2_(SO_4_)_3_ (Sigma Aldrich), ferric citrate FeC_6_H_5_O_7_ (Sigma Aldrich) were added at concentrations of 2.5, 5, 10 and 20 µM elementary iron. Splenocytes were cultured in RPMI-1640 medium (PAN Biotech) supplemented with 10% FCS (Biochrom), 2% sodium pyruvate (Sigma), 1× non-essential amino acids (Gibco), 0.01% β-mercaptoethanol (Roth), 1% penicillin/streptomycin (Lonza) and 2 mM L-glutamine (Lonza).

### BrdU Labeling of Splenocytes

Splenocytes were cultured as described before and pulsed with 10 µM BrdU (Sigma-Aldrich) 4 h before harvesting. Intracellular staining for BrDU with surface co-staining for CD3, CD4 and CD8 was performed with BrdU Flow Kit (BD) according to the manufacturers` instructions and cells were analyzed with flow cytometry. Iron sources ferric chloride FeCl_3_, ferric sulfate Fe_2_(SO_4_)_3_, ferric citrate FeC_6_H_5_O_7_ were added at indicated concentrations.

### T Cell Proliferation and Differentiation Assays

Total and naive CD4^+^ T cells were isolated using the MagniSort Mouse CD4 T cell Enrichment Kit and MagniSort Mouse CD4 Naive T cell Enrichment Kit (Invitrogen), respectively. 5 × 10^5^ cells in 200 µl RPMI-1640 medium (PAN Biotech) supplemented with 10% FCS (Biochrom), 2% sodium pyruvate (Sigma), 1× non-essential amino acids (Gibco), 0,01% β-mercaptoethanol (Roth), 1% penicillin/streptomycin (Lonza) and 2 mM L-glutamine (Lonza) were cultivated in 96-well U-bottom cell culture plates (Greiner) coated with 4 µg/ml anti-CD3 (BD Pharmingen) and supplemented with 1 µg/ml anti-CD28 (BD Pharmingen). For differentiation of the naive CD4^+^ T cells into Th1 lymphocytes, the culture was additionally supplemented with 10 ng/ml mIL-12 (Invitrogen) and 5 μg/ml anti-IL-4 (Invitrogen). Th2 cells were differentiated with 10 ng/ml IL-4, 5 µg/ml anti-IL-12, and 5 µg/ml anti-IFNγ. For Th17 differentiation 5 ng/ml TGFβ, 40 ng/ml IL-6, 10 ng/ml IL-23, 2 µg/ml anti-IFNγ, and 2 µg/ml anti-IL-4 were added to the medium, for regulatory T cell differentiation 5 ng/ml TGFβ, 20 ng/ml IL-2, 5 µg/ml anti-IL-12, 5 µg/ml anti-IFNγ, and 5 µg/ml anti-IL-4 were used. Iron sources ferric cloride FeCl_3_, ferric sulfate Fe_2_(SO_4_)_3_, and ferric citrate FeC_6_H_5_O_7_ were added at a concentration of 5 µM for 48 h.

### RNA Extraction and Quantitative Real-Time PCR

Total RNA was prepared from nitrogen-frozen tissues with peqGOLD Tri-Fast™ (Peqlab). For reverse transcription 4 µg RNA was used. Real-time PCR was performed on a CFX96 light cycler (Bio-Rad) using Ssofast Probes Supermix and Ssofast EvaGreen Supermix (Bio-Rad Laboratories GmbH). Relative gene expression was calculated with the ΔΔCT method, normalizing the results to the value for the Hypoxanthine phosphoribosyltransferase (*Hprt*) gene. *Havcr2 (Tim-3)* fw 5`-atgtgactctggatgaccatggga-3`; rv 5`-agtgaccttggctgctttgatgtc-3`; probe 5`-aggtcactccagctcagactgcccat-3`; *Hprt* fw 5`-gaccggtcccgtcatgc-3`, rv 5`-tcataacctggttcatcatcgc-3`, probe 5`-acccgcagtcccagcgtcgtc-3`; *TfR-1/CD71* fw 5`-atgaggaaccagaccgttatg-3`, rv 5`-ccccaagtttcaactgacc-3`, prob 5`-cccacactggacttcgccgca-3`. *DMT-1* fw 5`-ggactgtggacggtcggtaa-3`, rv 5`-aatgttgccaccgctggt-3`, probe 5`-catctcgaaagtcctgctgaccga-3`, *ZIP14* fw 5`-attgccctagccgatatgttc-3`, rv 5`-tgccctgaatacattgtgagg-3`.

### Statistics

Statistical analysis was generated using Prism GraphPad software (Version 7). Significance was determined by unpaired two-tailed t test to assess data, when only two groups were compared. For multiple comparisons Analysis of variance (ANOVA) combined with Tukey`s post test was performed. P values less than 0.05 were considered as statistically significant in any test.

### Specific Statistical Data Analyzed in Main Figures

**Figure 1:**
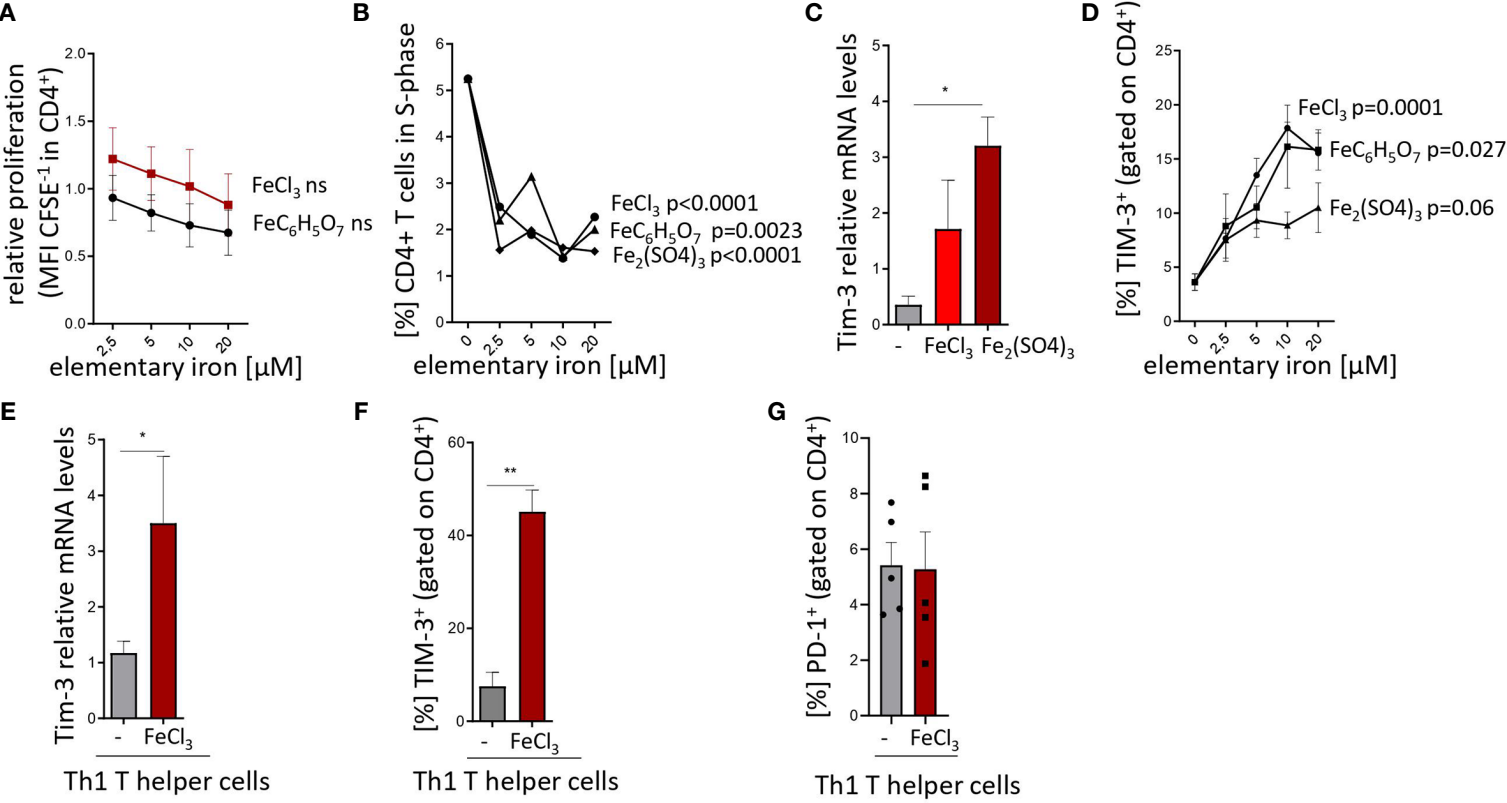
Iron inhibits T helper cell priming and expansion and stimulates TIM-3 expression in pan-CD4^+^ T cells and Th1 lymphocytes. **(A, B)** Splenocytes from C57Bl/6 male mice were stimulated with plate-bound anti-CD3 antibodies and FeCl_3_, FeC_6_H_5_O_7_, Fe_2_(SO_4_)_3_ were added at different concentrations. Proliferation of CD4^+^ T cells was measured as CFSE dilution **(A)** and BrdU incorporation **(B)** 72 h after culture start by flow cytometry. **(C, D)** Isolated CD4^+^ T cells were stimulated with plate-bound anti-CD3 and soluble anti-CD28 and 5 µM **(C)** or indicated concentrations **(D)** of FeCl_3_, FeC_6_H_5_O_7_, or Fe_2_(SO_4_)_3_. *Tim3* transcript levels were determined by quantitative real-time PCR and normalized to *Hprt* mRNA levels using the ΔΔCT method **(C)**. Percentage of TIM3-positive cells was measured by flow cytometry **(D)**. **(E–G)** Splenic naive CD4^+^ lymphocytes were differentiated to Th1 cells by stimulation with plate bound anti-CD3, soluble anti-CD28, anti-IL-4 antibodies and IL-12 with or without (−) 5 µM FeCl_3_ for 72 h. *Tim3* transcript levels were determined by quantitative real-time PCR and normalized to *Hprt* mRNA levels using the ΔΔCT method (**E**). Percentages of TIM-3 **(F)** and PD-1 **(G)** positive cells were measured by flow cytometry. Means ± SEM are shown in the plots. Statistical significance was assessed by one-way ANOVA for each iron source **(A, B, D)** and by two-tailed Student`s t-test **(C, E, F, G)**. Results of T test and ANOVA are presented in the plots. *p < 0.05, **p < 0.01. **(A–F)** n = 3. **(G)** n = 5.

**Figures 1A, B, D**: one-way ANOVA for particular iron forms, ANOVA p values presented in the plots.

**Figures 1C, E, F, G**: Two-tailed T test for control – iron comparisons, p values presented in the plots. **(C)** p = 0.034, **(E)** p = 0.044, **(F)** p = 0.006, **(G)** ns.

**Figure 2:**
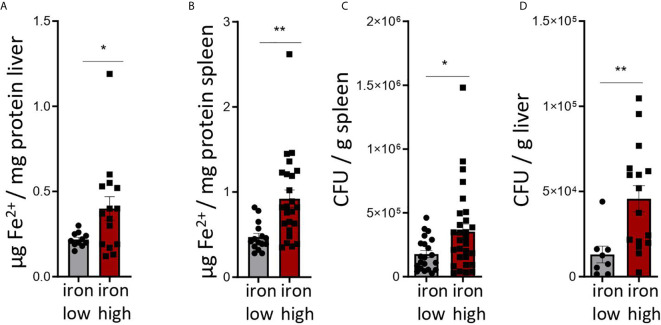
Dietary iron supplementation increases iron stores and compromises anti-bacterial host defense in the chronic *Salmonella Typhimurium* infection. C57BL/6 male mice expressing functional Nramp^G169^ were fed either a high iron (5 g iron/kg diet) or a low iron (≤9 mg iron/kg diet) diet two weeks before and during infection with 500 CFU of *S*. *Typhimurium*. The animals were analyzed 14 days post infection. **(A, B)** Iron content of the liver **(A)** and spleen **(B)** was assessed by a colorimetric assay and normalized to the protein content of the organ homogenates. **(C, D)** Bacterial burden was determined by plating of spleen **(C)** and liver **(D)** homogenates and CFU counting. CFU numbers were normalized to organ mass. Means ± SEM are shown in the plots. Statistical significance was determined by the two-tailed Student`s t-test. *p < 0.05, **p < 0.01. **(A)** iron low n = 11, iron high n = 15; **(B)** iron low n = 17, iron high n = 24; **(C)** iron low n = 21, iron high n = 29; **(D)** iron low n = 8, iron high n = 16.

**Figure 2**: Two-tailed T test for low iron–high iron comparisons, p values presented in the plots. **(A)** p = 0.038, **(B)** p = 0.002, **(C)** p = 0.022, **(D)** p = 0.009.

**Figure 3:**
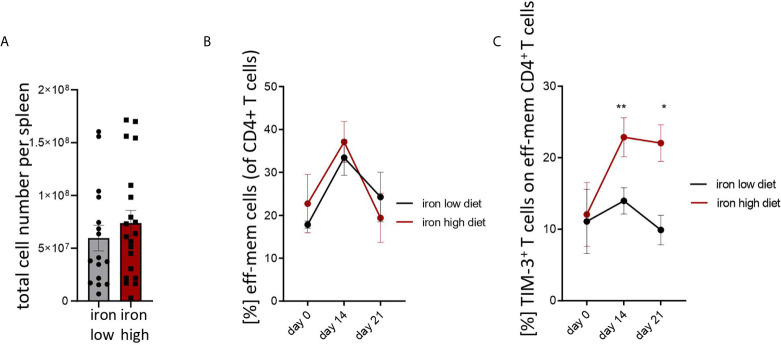
Iron loading increases surface TIM-3 protein on effector/memory T helper cells during *Salmonella typhimurium* infection. Nramp^G169^-expressing C57Bl/6 male mice were fed either a high iron (5 g iron/kg diet) or a low iron (≤9 mg iron/kg diet) diet two weeks before and during infection with 500 CFU of *S*. *Typhimurium*. **(A)** Total spleen cellularity was measured by flow cytometry on day 14 post infection. **(B, C)** Percentages of effector/memory T helper cells (CD4^+^CD62L^lo^CD44^hi^) within CD4^+^ lymphocytes and percentage of TIM3-positive cells within the effector/memory T helper population were quantified by flow cytometry at the indicated time points. Means ± SEM are shown in the plots. Statistical significance was determined by two-tailed T test **(A)** and two-way ANOVA with Tukey post-hoc tests **(B, C)**. Results of T test and post-hoc tests are presented in the plots. *p < 0.05, **p < 0.01. **(A)** iron low n = 16, iron high n = 22. **(B)** day 0 low iron n = 4, high iron n = 4; day 14 low iron n = 12, high iron n = 12; day 21 low iron n = 3, high iron n = 5. **(C)** day 0 low iron n = 4, high iron n = 4; day 14 low iron n = 9, high iron n = 11; day 21 low iron n = 3, high iron n = 5.

**Figure 3**: Two-tailed T test for low iron–high iron comparisons, p values presented in the plots. **(A)** ns, **(B)** ns, **(C)** day 14 p = 0.0018, day 21 p = 0.018.

**Figure 4:**
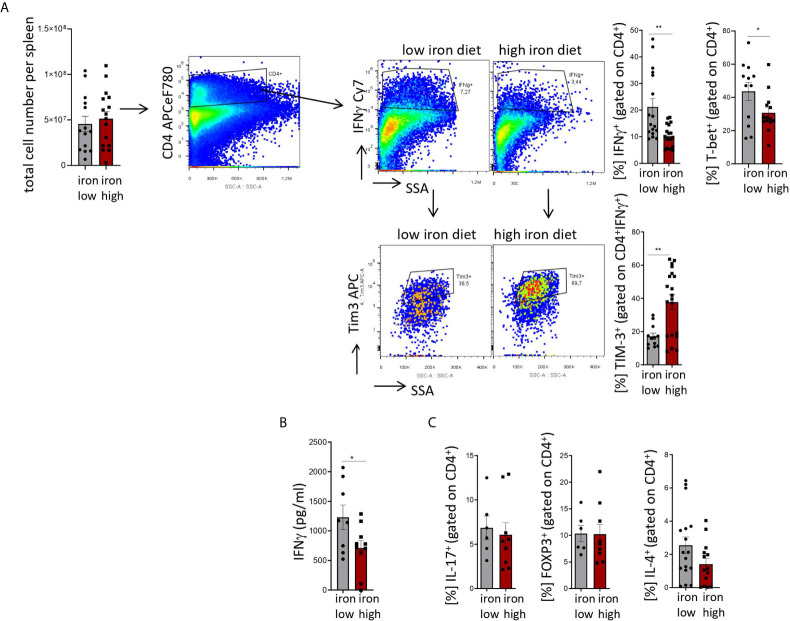
Stimulation of TIM-3 expression and impaired differentiation of Th1 cells by iron loading in *Salmonella typhimurium* infection. Nramp^G169^-expressing C57Bl/6 male mice were fed either a high iron (5 g iron/kg diet) or a low iron (≤9 mg iron/kg diet) diet two weeks before and during infection with 500 CFU of *S*. *Typhimurium*. The animals were analyzed on day 14 post infection. **(A)** Total number of cells per spleen, percentages of splenic Th1 cells (IFNγ^+^, T-bet^+^) within CD4^+^ helper T cells and percentages of TIM-3 positive cells within the Th1 subset were quantified by flow cytometry. **(B)** Serum concentration of IFNγ was measured by a multiplex assay. **(C)** Percentages of Th17 (IL-17A^+^), regulatory T helper T cells (FOXP3^+^), and Th2 (IL-4^+^) within the CD4^+^ helper T lymphocyte subset were determined by flow cytometry. Means ± SEM are shown in the plots. Statistical significance was assessed by two-tailed T test. *p < 0.05, **p < 0.01. **(A)** IFNγ ^+^ iron low n = 17, iron high n = 19; TIM3^+^ iron low n = 12, iron high n = 20; **(B)** iron low n = 8, iron high n = 10. **(C)** IL-17^+^ iron low n = 6, iron high n = 9; FOXP3^+^ iron low n = 6, iron high n = 9; IL-4^+^ iron low n = 17, iron high n = 14.

**Figure 4**: Two-tailed T test for low iron–high iron comparisons, p values presented in the plots. **(A)** %IFNγ^+^ p = 0.001, %Tim-3^+^ p = 0.002, **(B)** p = 0.041, **(C)** ns.

**Figure 5:**
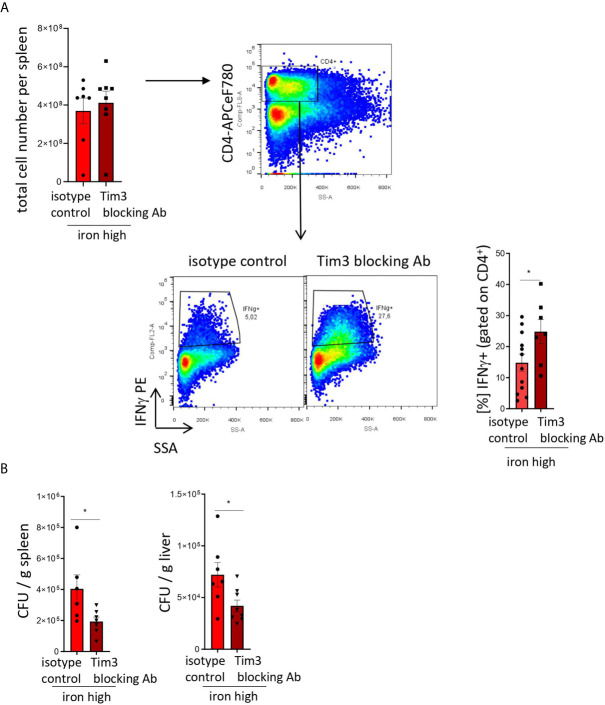
TIM-3 blockade restores Th1 cell differentiation and pathogen control in iron supplemented *S. Typhimurium*-infected mice. Nramp^G169^-expressing C57Bl/6 male mice were fed a high iron (5 g iron/kg diet) diet two weeks before and during infection with 500 CFU of *S*. *Typhimurium*. From the day of infection on, mice were intraperitoneally injected with 100 µg *in vivo* MAb anti-mouse TIM-3 antibody or *in vivo* MAb rat IgG2a isotype control on every second day. The animals were analyzed on day 14 post infection. **(A)** Total cell number per spleen and percentages of Th1 (IFNγ ^+^) cells within the CD4^+^ helper T cells were assessed by flow cytometry. **(B)** Bacterial burden was determined by plating of spleen and liver homogenates and CFU counting. CFU numbers were normalized to organ mass. Means ± SEM are shown in the plots. Statistical significance was assessed by two-tailed T test. *p < 0.05, **p < 0.01. **(A)** isotype control n = 12; Tim-3 blocking Ab n = 7. **(B)** spleen isotype control n = 6, Tim-3 blocking Ab n = 7; liver isotype control n = 7, Tim-3 blocking Ab n = 8.

**Figure 5**: Two-tailed T test for low iron–high iron comparisons, p values presented in the plots. **(A)** p = 0.048, **(B)** CFU/g spleen p = 0.037, CFU/g liver p = 0.033.

## Results

### Iron Supplementation Inhibits T Cell Priming and Expansion and Upregulates the Immune Checkpoint Regulator TIM-3

Based on previous observations that iron loading affects Th1 and Th2 cell differentiation (36, 37) and the regulatory effects of TIM proteins on T cell priming (38) we hypothesized a possible interconnection between those pathways.

First, iron supplementation of anti-CD3-primed splenocytes isolated from naive mice induced a slight decrease in the proliferation and dramatically slowed cell cycle progression of T cells as demonstrated by CFSE dilution (**Figure 1A**) and BrdU pulsing (**Figure 1B**) assays even at concentrations as low as 2.5 to 5 µM (39). Both effects were independent of the iron source (chloride, sulfate and citrate) and, hence, independent of iron source-inherent differences in solubility, dissociation properties, and cellular iron availability. In parallel, we found a strong upregulation of both transcript and surface protein levels of TIM-3 in the total CD4^+^ T cell fraction primed with anti-CD3/CD28 and supplemented with iron, as compared to control-treated cultures (**Figures 1C, D**). Again, this upregulation was observed with different iron supplements and occurred in a dose-dependent manner. Notably, the same mode of regulation of *Tim-3* mRNA and protein levels was observed in naive Th cells differentiated into Th1 lymphocytes in presence of iron (**Figures 1E, F**). Of note, surface abundance of another checkpoint regulator, PD-1, was comparable in control- and iron-stimulated Th1 differentiation cultures (**Figure 1G**).

### *In Vivo* Dietary Iron Supplementation Impairs Pathogen Control, Upregulates TIM-3 and Reduces Th1 Differentiation

We then investigated if the strong inhibitory effects of iron on T cell priming and the stimulation of the checkpoint regulator TIM-3 can be recapitulated *in vivo* and if this affects anti-bacterial defense in a model of chronic bacterial infection.

To this end we used C57BL/6 mice with transgenic expression of a functional natural resistance associated macrophage protein 1 (NRAMP1 or SlC11A1), which results in an improved host resistance to infections with *S. Typhimurium* and thus prolonged bacterial infection allowing to study T cell responses over time (**Figure 2**, **Supplementary Figure 1**).

Mice were fed with low (<9 mg elementary Fe/kg) (26) or high iron (5 g/kg) diets for two weeks and during the course of the infection. After intraperitoneal infection with 500 CFU of *S*. *Typhimurium* (27–29) mice were followed up for 14 days. Since the canonical high iron feeding regime used in the short-term Salmonella infection model (25 g/kg) led to strong weight reduction and pre-term mortality, we decided to induce iron overload with chow containing less iron (5 g Fe/kg), which was sufficient to significantly increase the levels of the metal in the canonical storage organs, liver and spleen, as compared to mice on a low-iron diet (**Figures 2A, B**). This was accompanied by elevated bacterial burden in those organs (**Figures 2C, D**; **Supplementary Figure 1**). In line with the *in vitro* results, TIM-3 surface protein expression in effector-memory T helper cells (CD4^+^CD62L^lo^CD44^hi^), which is the population primed and expanded during infection (40), was progressively increasing over time (**Figure 3C**). Of note, neither spleen cellularity (**Figures 3A**, **4A**), nor general abundance of this CD4^+^ T cell subset was changed by dietary iron supplementation (**Figure 3B**). The IFNγ expressing Th1 subset plays a central role in mounting effective immune protection against *S. Typhimurium* (40). Dietary iron loading strongly upregulated surface TIM-3 levels in that subset (**Figure 4A**, **Supplementary Figure 1D**) and dramatically reduced frequencies of Th1 cells within splenic helper CD4^+^ and CD3^+^ pan-T cells on day 14 post bacteria challenge. This suggested reduced priming and differentiation of Th1 cells and/or their exhaustion by high iron availability (**Figure 4A**, **Supplementary Figure 1C**). In line with the reduced Th1 differentiation, we found significantly decreased percentages of helper T cells expressing T-bet, the key transcription factor orchestrating development of the Th1 phenotype (**Figure 4A**). Consequently, at the systemic level, the diminished Th1 cell expansion culminated in reduced circulating concentration of IFNγ in iron-fed mice (**Figure 4B**). Of note, we did not observe any substantial effects of dietary iron overload on the residual, self-sustaining pool of Th1, Th2, Th17 and Treg lymphocytes in the spleen (**Supplementary Figure 4**).

Analogically to the *in vitro* setting, iron loading *in vivo* had no impact on the levels of another immune checkpoint protein, PD-1, on the surface of helper T lymphocytes (**Supplementary Figure 1E**), which suggests that the inhibitory effects of the metal are limited to TIM-3 regulation. Importantly, the effects of iron were highly Th1 cell specific, as neither the IL-4^+^ Th2, nor the IL-17A^+^ Th17, nor the regulatory FOXP3^+^ T cell subsets displayed numerical alterations upon high-iron diet (**Figure 4C**). In addition, serum levels of IL-2, the key cytokine for T cell expansion were not changed after iron loading of *S. Typhimurium* infected mice (**Supplementary Figure 2A**). Of note, the levels of major inducers of TIM-3 expression, namely IL-12 (31) and IL-27 (32), were not significantly affected by iron challenges (**Supplementary Figures 2B, C**), suggesting that iron controls TIM-3 mRNA and protein expression rather directly *via* a cell-intrinsic mechanism and not *via* modulating the expression of those two TIM-3 inducing cytokines.

So far, our data clearly showed the negative impact of iron overload on Th1 cell differentiation, function, and microbial control. However, in theory these effects could also be attributed to the particularly effective T cell response in the low iron setting or to the extreme difference in iron content between the diets. To account for that, we performed the *S. Typhimurium* infection experiments in animals fed the low iron (<9 mg/kg), standard (166 mg/kg) and high iron chow (5 g/kg). As shown in **Supplementary Figure 5**, there was no significant difference neither in Th1 (CD4^+^ IFNγ^+^) differentiation or expression of surface TIM-3 in Th1 cells (A, B), nor in abundance of Th2, Treg and Th17 cells (C–E) between the low- and standard iron chow. Conversely, the Th1 cell percentages were significantly higher and their TIM-3 expression was significantly lower in the animals fed standard diet than in iron overloaded mice (**Supplementary Figures 5A, B**).

### Dietary Iron Supplementation Has No Effect on Numbers of Non-Lymphoid Immune Cells in Response to *S. Typhimurium* Infection

Because TIM-3 is not only expressed on lymphocytes but also on myeloid cells such as monocytes and macrophages (41), we reasoned that innate immune cell numbers may also be affected by iron challenge. As shown in **Supplementary Figure 3**, we could not find any significant differences in relative frequencies of any of the investigated myeloid populations (neutrophils, macrophages, Ly6C^hi^ classical monocytes and Ly6C^lo^ resident monocytes) attributed to the iron content of the diets after 14 days of infection with *S. Typhimurium*. These results point towards comparable levels of systemic inflammation in both experimental groups. The percentages of TIM-3-expressing cells in the myeloid leukocytes were found, in general, to be less than half of the expression of TIM-3 in the Th1 lymphocytes and, in addition, were not significantly affected by dietary iron loading (**Supplementary Figure 3**, **Figure 4A**).

### Administration of TIM-3 Blocking Antibodies Restores Th1 Cell Expansion and Improves Pathogen Control in Iron-Supplemented Mice

We next tested whether neutralization of TIM-3 affects iron induced Th1 cell exhaustion and the impaired immune control of chronic *S. Typhimurium* infection.

To test that, we administered isotype and blocking antibodies against TIM-3 (17) to iron loaded animals infected with *S*. *Typhimurium*. In this setting, blocking TIM-3 significantly increased the numbers of IFNγ-producing Th1 lymphocytes within the CD4^+^ T cell compartment as compared with animals receiving the isotype antibody (**Figure 5A**). Moreover, it virtually restored the Th1 differentiation to the levels observed in *Salmonella*-infected mice fed an iron-low chow (**Figure 4A**). Importantly, this intervention had no effect on the total cellularity of the spleens (**Figure 4A**). Furthermore, the TIM-3 blockade *in vivo* improved bacterial control as demonstrated by a significant reduction of bacterial burden in the liver and spleen (**Figure 5B**). Collectively, our findings demonstrate that TIM-3 links the negative effects of iron on IFNγ production by Th1 lymphocytes to impaired host defense against the intracellular pathogen *S. Typhimurium*.

### Th1 Cells Express the Highest Levels of Cellular Iron Importers Among the Th Subsets

Finally, we sought to investigate the mechanism behind the highly specific TIM-3-mediated impact of iron on Th1 lymphocytes but not on the other CD4^+^ T cell subsets. The most obvious explanation is a potential difference in uptake of iron by various Th cell classes. To test that, we investigated levels of cellular iron importers in Th1, Th2, Th17, and Treg lymphocytes. Iron can be ingested by the cell in two forms: as transferrin-bound iron (TBI), being the physiological form of the element, and as chemically reactive, potentially toxic non-transferrin-bound iron (NTBI), as for example upon iron overload conditions. Interestingly, among the Th subsets differentiated *ex vivo*, Th1 cells demonstrated the highest mRNA and surface protein expression of both the TBI importer TfR1 (transferrin receptor 1) and NTBI uptake proteins DMT1 (di-valent metal transporter 1), as well as ZIP14 (Zrt- And Irt-Like Protein 14) (**Supplementary Figure 6**). This may be related to the superior iron uptake capabilities of Th1 cells and the higher sensitivity to iron compared with other CD4^+^ T cells.

## Discussion

Here we report a novel mechanism by which the increased availability of iron leads to an unfavorable outcome of infections with the intracellular bacterium *Salmonella enterica serovar Typhimurium*. Iron specifically acts on IFNγ-producing CD4^+^ T cells and stimulates the expression of the negative regulatory surface receptor or ‘immune checkpoint’ inhibitor TIM-3, thus reducing the functionality of these cells. This is in line with earlier studies indicating that Th1 T cells are exquisitely sensitive to iron perturbations because intracellular iron depletion affects Th1 cells more than the Th2 cell subset (8, 36, 37). In our model of chronic bacterial infection, the effects of iron were highly specific for Th1 cells as neither the number of Th2, nor Th17 or Treg subsets displayed differences after high iron diet. Interestingly, our previous report provided evidence that the function of the CD8^+^ IFNγ-producing T cell subset in murine mammary carcinoma was strongly hampered by intravenous iron supplementation (39).

Principally, iron can be ingested by cells as transferrin-bound iron, being the physiological form of the element, which is mediated by the TfR-1. Additionally, iron can be taken up as chemically reactive and potentially toxic non-transferrin-bound iron, mediated by the receptors DMT1 and ZIP14. This form of iron ingestion happens e.g. upon iron overload conditions. Our *in vitro* results showed a preferential expression of iron uptake receptors, both of the transferrin- and non-transferrin bound iron forms, in the Th1 subset which is likely to explain their particular sensitivity to iron overload. Appropriately, the CD4^+^ T cell subset was described to be the main effector subset in the defense of *Salmonella* infections (20, 40, 42).

Importantly, in chronic infections T cell exhaustion is described to develop as a state of cellular and immunological dysfunction as a consequence of persisting antigenic stimulation (12), leading to reduced cytokine production and over-expression of inhibitory receptors. TIM-3, an inhibitory receptor widely expressed on Th1 cells, is described as an important player in T cell dysfunction and exhaustion (12–15). This supports our finding that iron specifically impacts on TIM-3 expression in Th1 cells during chronic infection. Indeed, we showed that dietary iron loading strongly upregulated surface TIM-3 levels on Th1 cells. A pathophysiological role of this interaction is strongly supported by the finding that anti-TIM-3 treatment improved infection control in iron loaded mice to levels observed in animals on an ironbalanced diet. This is in line with recently published studies, demonstrating the crucial role of TIM-3 over-expression on T cells in the control of infections with intracellular bacteria such as *Mycobacterium tuberculosis*, where IFNγ mediated immune effector pathways play a decisive role (23, 43, 44). Accordingly, in *M. tuberculosis* infected mice administration of a TIM-3 fusion protein, acting as a molecular sink for TIM-3 ligands, reduced the bacterial burden (22). In our model, similar to the *M. tuberculosis* infection model, blocking TIM-3 overcame the detrimental effect of iron on IFNγ-producing Th1 cells. Furthermore, it rescued Th1 differentiation, and reduced bacterial burden in livers and spleens of *Salmonella* infected mice This highlights the role of TIM-3 as a crucial negative regulator of Th1 cell expansion and Th1-mediated bacterial host defense acting downstream of iron in chronic *Salmonella* infection.

Our findings raise the question how iron impacts Th1 immunity by influencing the expression of TIM-3. According to our *in vitro* data (**Figure 1**) iron dose-dependently increased TIM-3 expression, and blocked cell cycle progression and differentiation of Th0 to Th1 cells. Hence, two non-exclusive mechanisms may be proposed: first, interference of iron with the Th1-specific transcription factor network and second, signaling induced by reactive oxygen species, as postulated in our previous report on iron and anti-tumor CD8^+^ T cells (39). While iron did not affect the expression of TIM-3-inducing cytokines, IL-12 and IL-27 *in vivo*, it still could affect signals mediated by those cytokines *via* the IL-12 receptor or *via* T-bet, the master switch transcription factor of Th1 cells. This is supported by our data showing similar effects of iron on T-bet and on IFNγ expression in Th1 cells. This would be in a line with the description of T-bet as important regulator of TIM-3 on Th1 cells (45). In addition, iron may act on transcription factors other than T-bet such as c-Jun N-terminal kinases (JNK) (46) which, in turn, can activate several down-stream factors including c-Jun and SMAD Family Member 4 (Smad4) which are known to *trans*-activate TIM-3 expression (47, 48).

Our recently published data (39) indicate that high cellular iron content in CD8^+^ T cells induces accumulation of reactive oxygen species (ROS) in mitochondria and that treatment with a mitochondria-specific ROS scavenger could restore T cell priming even in the high iron setting. Whether a ROS-mediated mechanism including ferroptosis (49) or mitochondrial dysfunction (50) as a consequence of iron loading underly the TIM-3 up-regulation and functional impairment of Th1 cells in high iron-fed *Salmonella-*infected animals remains to be investigated.

Importantly, the increased expression of TIM-3 in response to iron overload is pathophysiologically relevant as we were able to restore host defense against *Salmonella* by a TIM-3 blocking antibody. As microbial resistance against major classes of antibiotics continues to increase, immune-modulatory therapies that strengthen host defence mechanisms and promote bacterial killing will become more and more important. Therefore, the selective acquired immune deficit that iron overload exerts on Th1 immunity could be partly overcome by a monoclonal antibody against TIM-3. This may be specifically relevant in subjects with iron overload on the basis of repeated transfusions in the course of hematological diseases or hemoglobinopathies. Hence, our study provides a novel rational for an immune-modulatory therapy by blocking the checkpoint molecule TIM-3.

## Data Availability Statement

The raw data supporting the conclusions of this article will be made available by the authors, without undue reservation.

## Ethics Statement

The animal study was reviewed and approved by BMWFW Austria.

## Author Contributions

PT participated in the study design, data collection and analysis, and drafted the manuscript. GK, SE, and NB participated in the data collection, MN participated in the data collection and revised the manuscript. AS and ED revised the manuscript. GW and CP-O participated in the study design, data collection, analysis, data interpretation, and manuscript preparation. All authors contributed to the article and approved the submitted version.

## Funding

MN is supported by a grant from the Austrian Research Fund FWF (P33062), GW by grants from the Christian Doppler Society and an ERA-NET grant by the FWF (EPICROSS, I-3321), and CP-O received support by the Austrian Cancer Society/Tirol (P17006). NB was supported by the FWF doctoral college project W1253 HOROS.

## Conflict of Interest

The authors declare that the research was conducted in the absence of any commercial or financial relationships that could be construed as a potential conflict of interest.

## Abbreviations

CFSE, carboxyfluorescein succinimidyl ester; Ctrl, control; CFU, colony forming unit; DSS, dextran sodium sulfate; DMT1, di-valent metal transporter 1; IL, interleukin; IFNγ, interferon gamma; NTBI, non-transferrin bound iron; TBI, transferrin bound iron; Tc, cytotoxic T cell, Th, helper T cell; Treg, regulatory T cell; *S. Typhimurium, Salmonella enterica* serovar *Typhimurium*; TNF, tumor necrosis factor; STAT1, signal transducer and activator of transcription 1; TfR-1, transferrin receptor 1; TGF, transforming growth factor; PD1, programmed cell death protein 1; CTLA-4, cytotoxic T-lymphocyte-associated Protein 4; Lag-3, Lymphocyte-activation gene 3; PDL-1, programmed cell death 1 ligand 1; TIGIT, T cell immunoreceptor with Ig and ITIM domains; ZIP14, Zrt- And Irt-Like Protein 14.
