# Supplementary figures and images for: Regulation of Th1 T Cell Differentiation by Iron via Upregulation of T Cell Immunoglobulin and Mucin Containing Protein-3 (TIM-3)
Source: Front Immunol. 2021 May 24;12:637809. doi: 10.3389/fimmu.2021.637809 (PMC8181170; doi:10.3389/fimmu.2021.637809)

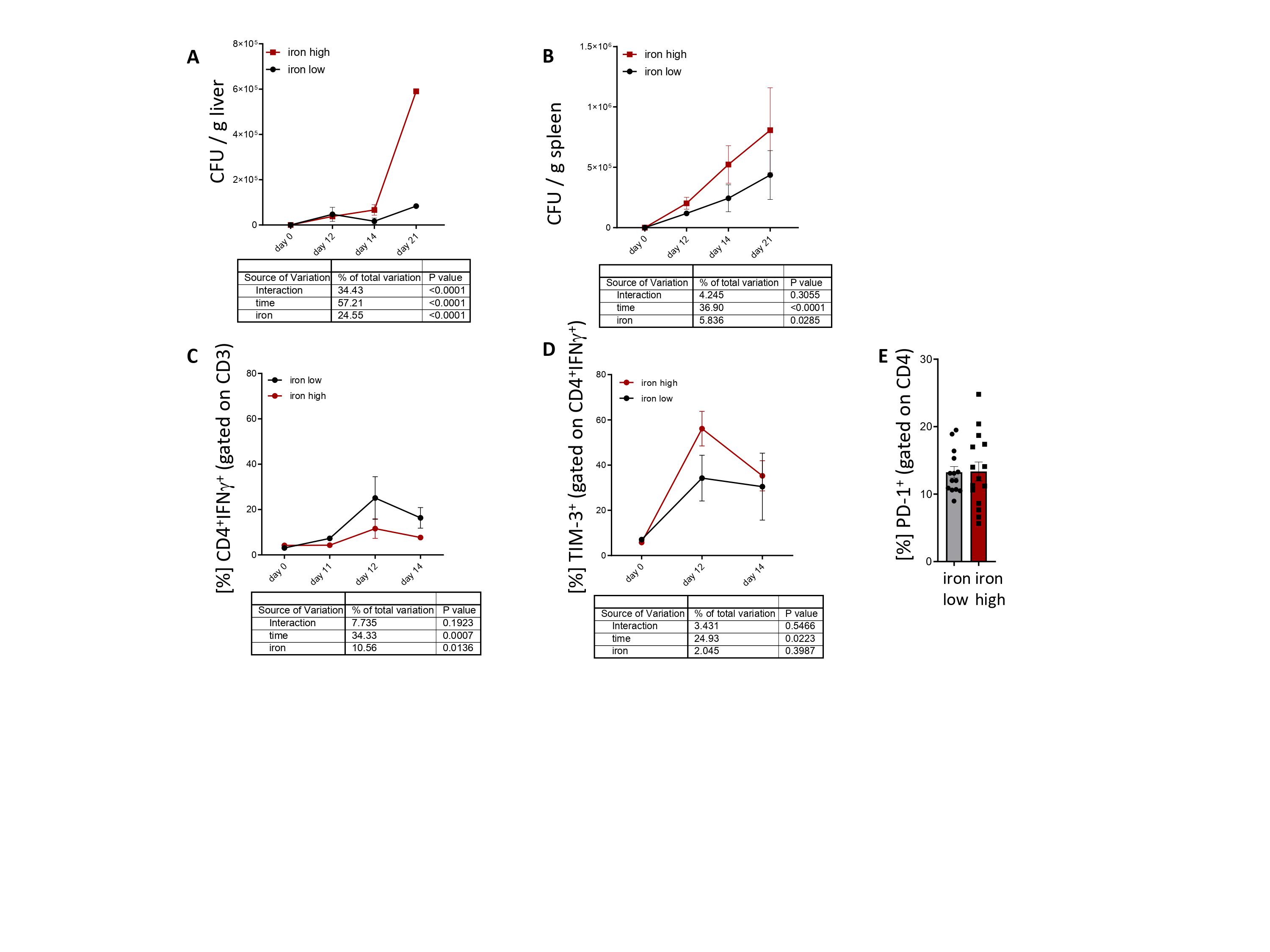

Supplement: Supplementary file 1 [file Image_1.jpeg]

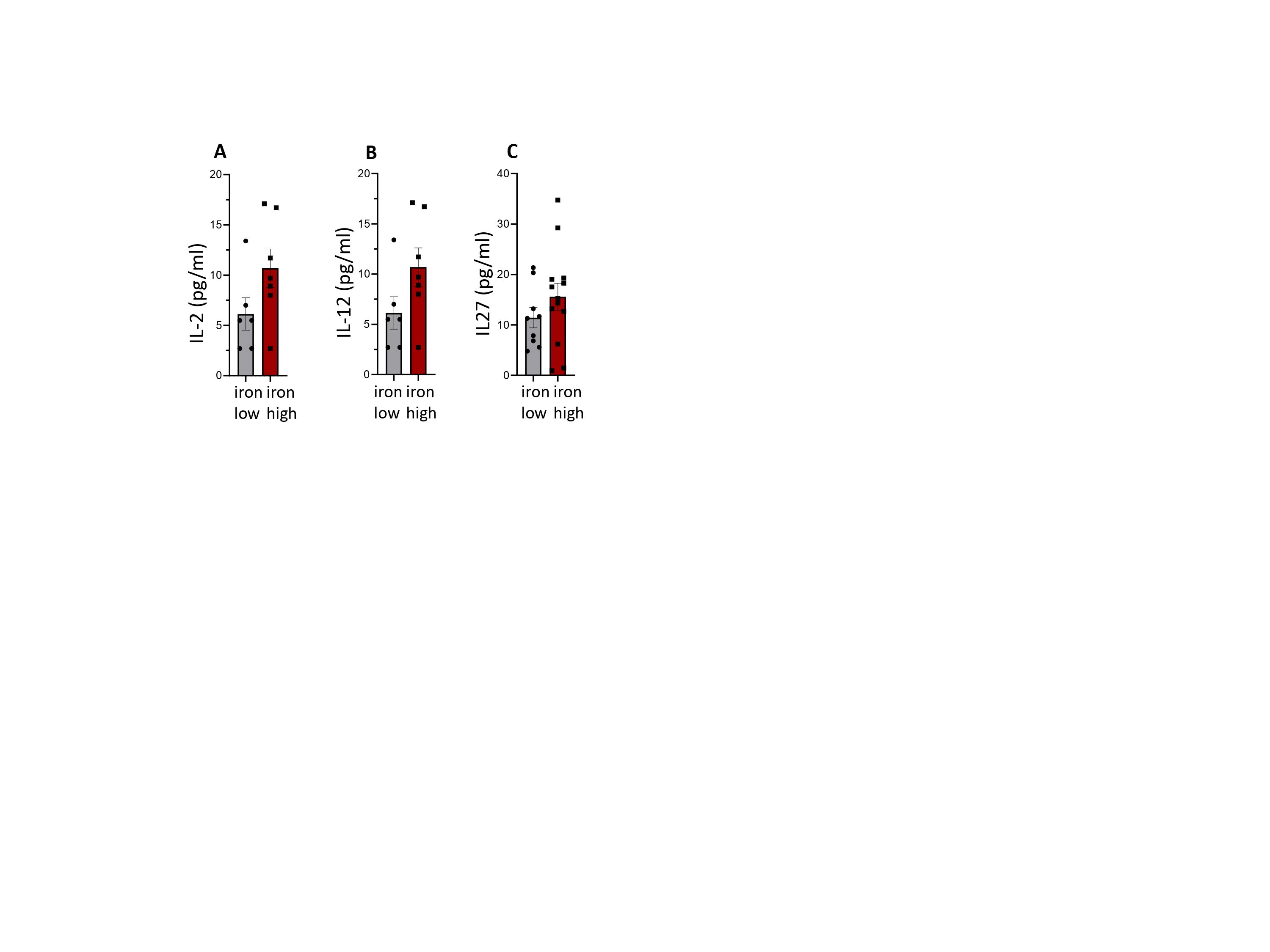

Supplement: Supplementary file 2 [file Image_2.jpeg]

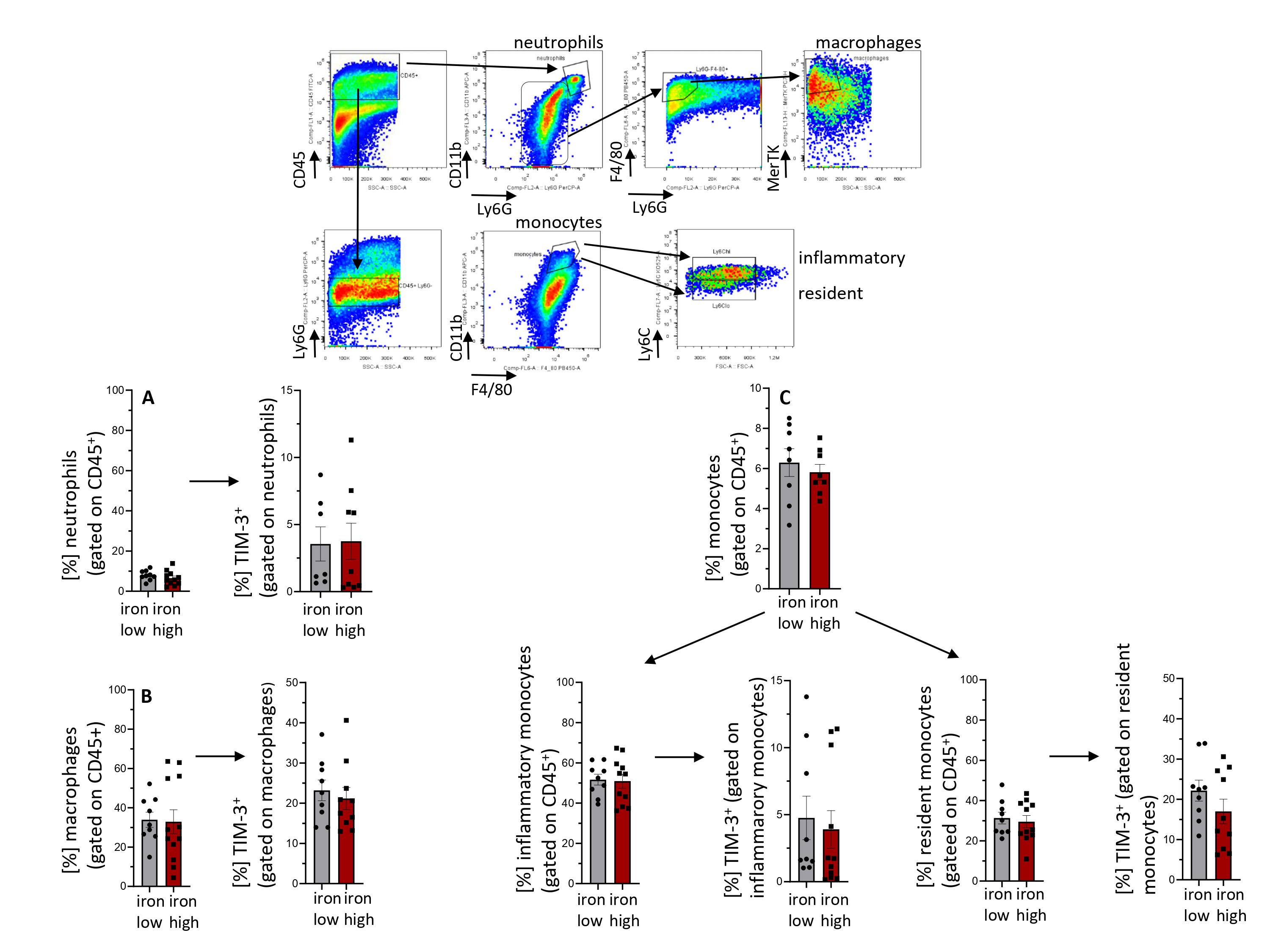

Supplement: Supplementary file 3 [file Image_3.jpeg]

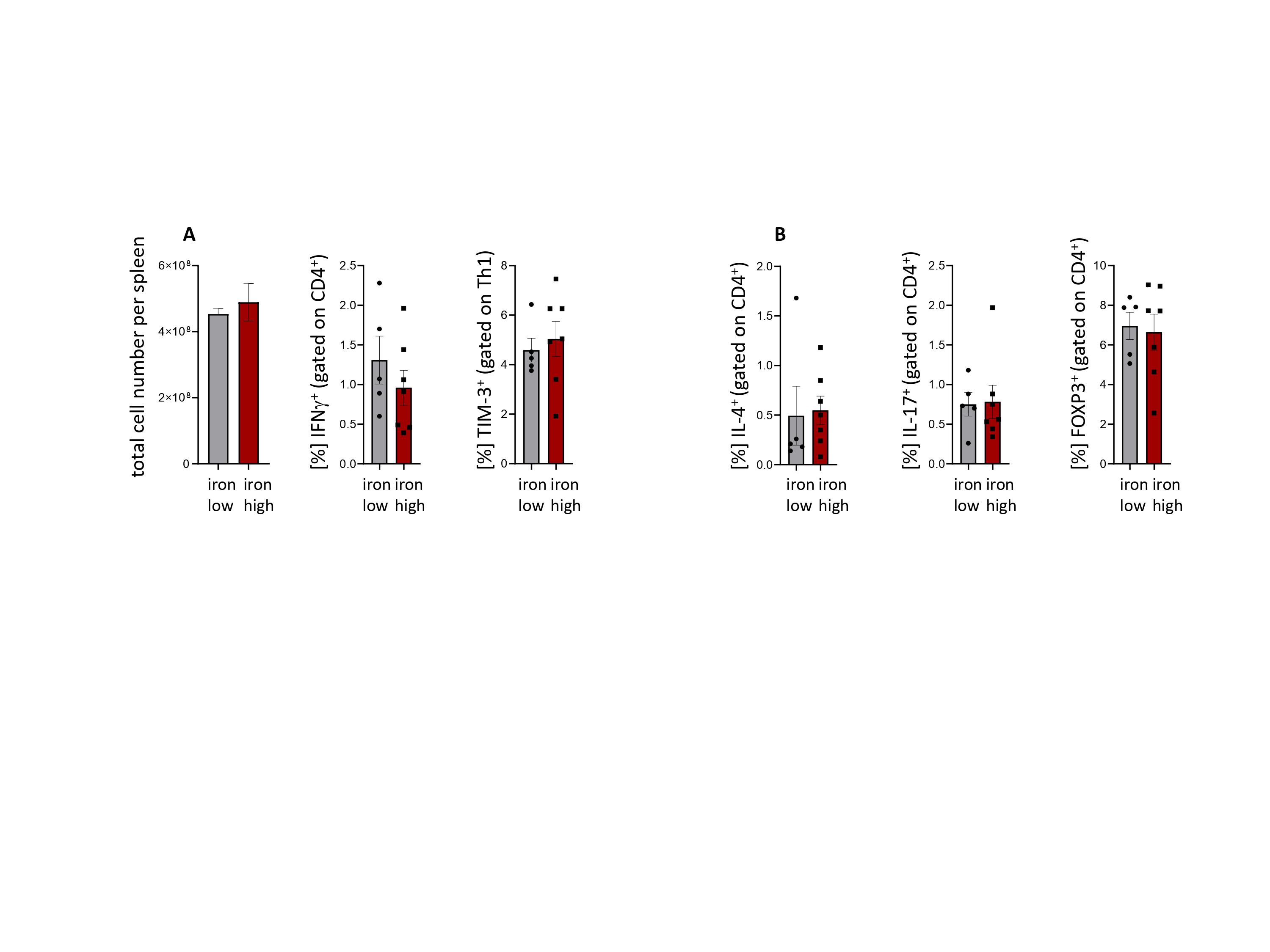

Supplement: Supplementary file 4 [file Image_4.jpeg]

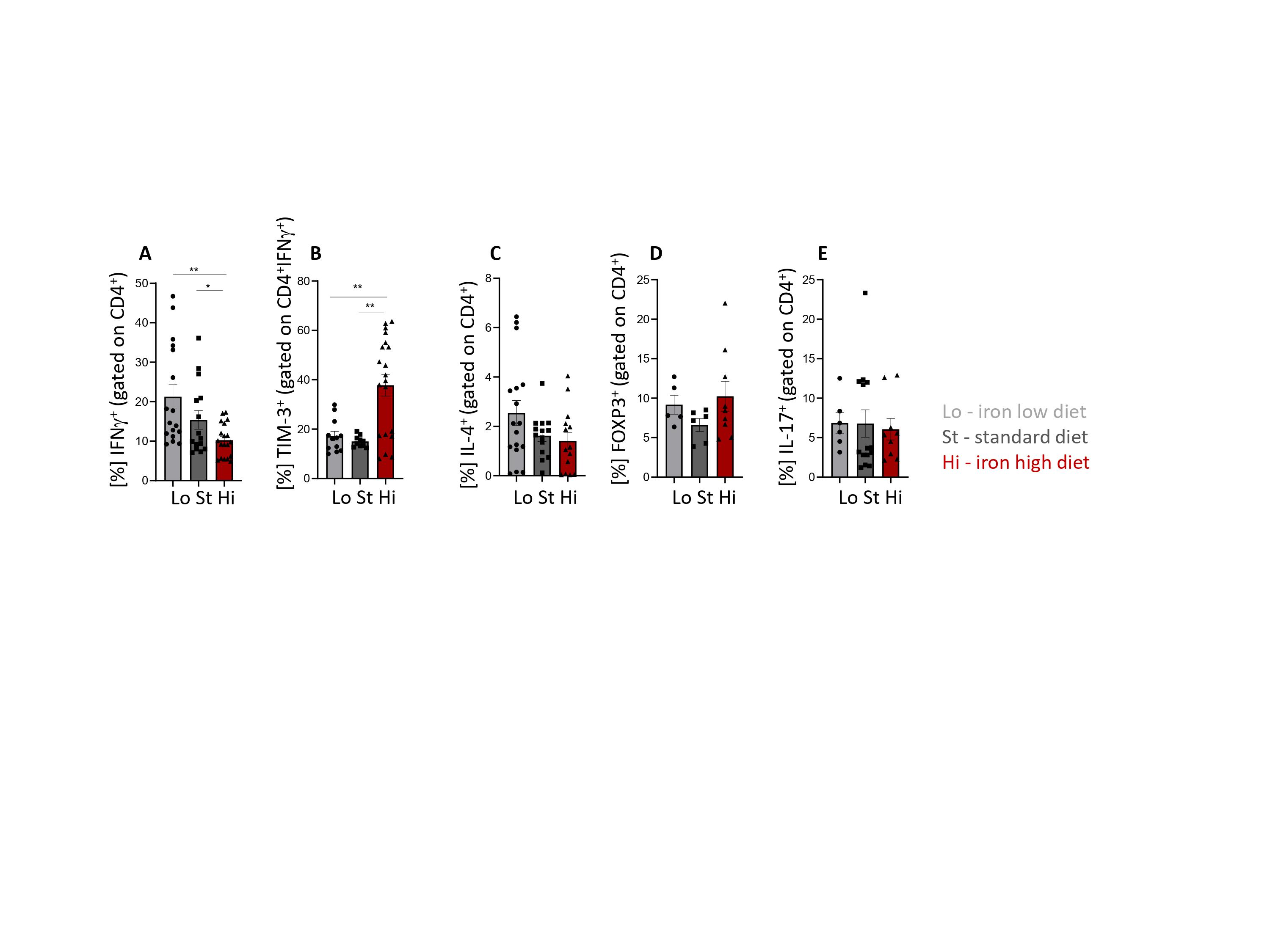

Supplement: Supplementary file 5 [file Image_5.jpeg]

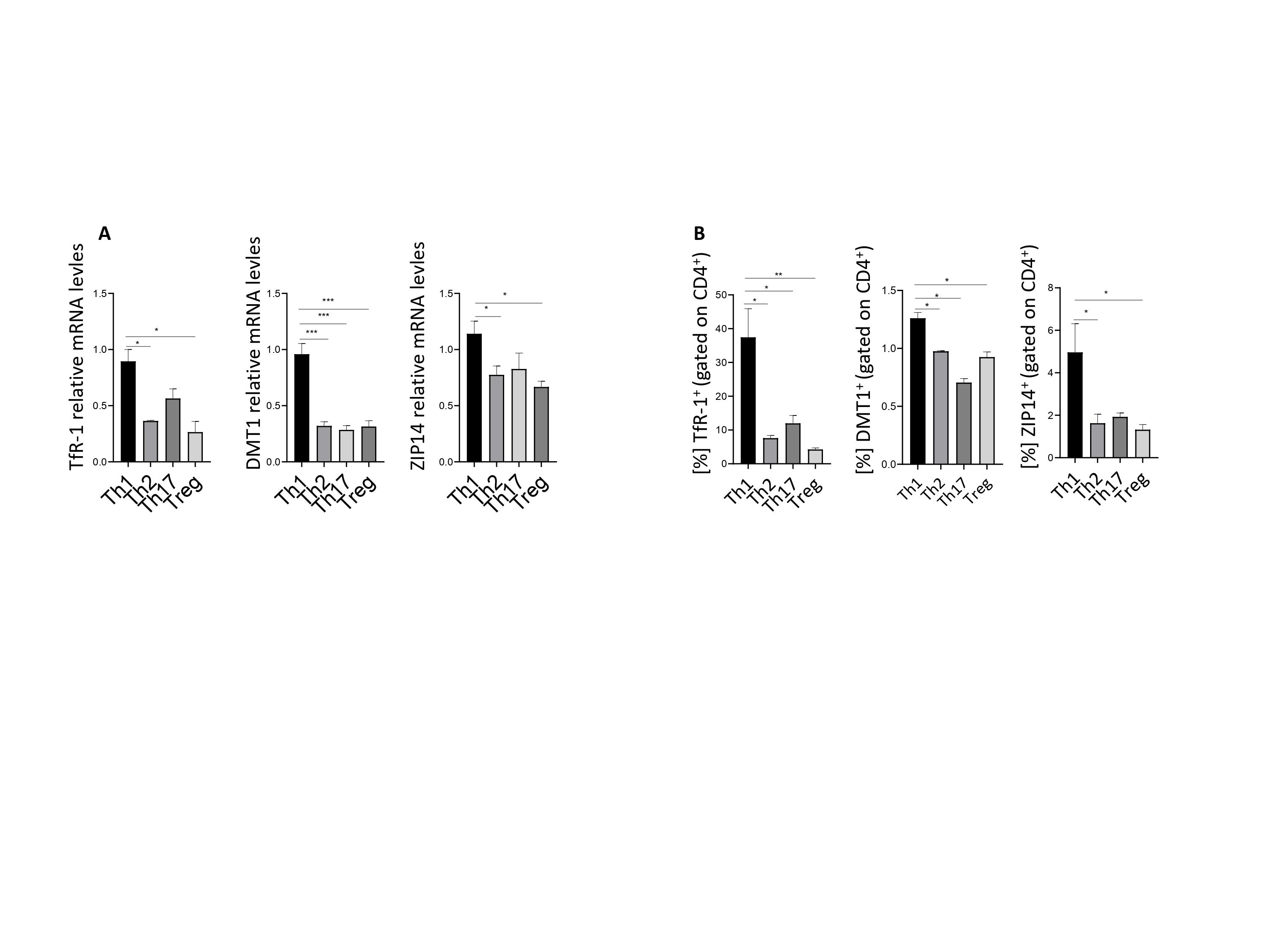

Supplement: Supplementary file 6 [file Image_6.jpeg]
